# Benefit of adjuvant chemotherapy in patients with T4 UICC II colon cancer

**DOI:** 10.1186/s12885-015-1404-9

**Published:** 2015-05-20

**Authors:** Andreas Teufel, Michael Gerken, Janine Hartl, Timo Itzel, Stefan Fichtner-Feigl, Christian Stroszczynski, Hans Jürgen Schlitt, Ferdinand Hofstädter, Monika Klinkhammer-Schalke

**Affiliations:** 1 Department of Medicine I, University of Regensburg, Regensburg, Germany; 2 Tumor Center, University of Regensburg, Regensburg, Germany; 3 Department of Surgery, University of Regensburg, Regensburg, Germany; 4 Department of Radiology, University of Regensburg, Regensburg, Germany

**Keywords:** Colon cancer, Colorectal cancer, Adjuvant therapy, T4 stage, UICC II

## Abstract

**Background:**

Colorectal cancer is the third most common cancer and a major cause of morbidity and mortality worldwide. Adjuvant chemotherapy is considered the standard of care in patients with UICC stage III colon cancer after R0 resection. Adjuvant therapy was not shown to be beneficial in patients with UICC stage II colon cancer. However, there is an ongoing discussion as to whether adjuvant chemotherapy may be beneficial for a subgroup of UICC II patients in a “high-risk situation” (such as T4).

**Methods:**

We investigated a Bavarian population-based (2.1 million inhabitants) cohort of 1937 patients with UICC II CRC treated between 2002 and 2012 in regard of the benefit of adjuvant chemotherapy for large (T4) tumors. Patients older than 80 years of age were excluded. Of 1937 patients, 240 had a T4 tumor (12 %); 77 of all T4 patients received postoperative chemotherapy (33 %). Kaplan-Meier analysis and Cox regression models were used for survival analyses.

**Results:**

Patients with a T4 tumor who received postoperative chemotherapy had a highly significant survival benefit in respect of overall survival (*p* < 0.001) and recurrence-free survival (*p* = 0.008). However, no difference was observed between oxaliplatin-containing and non-oxaliplatin-containing treatment regimens. G2 and G3 tumors were found to particularly benefit from adjuvant treatment. Chemotherapy, age at diagnosis, and tumor grading remained independent risk factors in the multivariate cox regression analysis.

**Conclusion:**

Our retrospective study demonstrated the significant benefit of adjuvant chemotherapy in the T4 subgroup of patients with UICC II colon cancer. Our data suggest that adjuvant chemotherapy should be seriously considered in these patients.

**Electronic supplementary material:**

The online version of this article (doi:10.1186/s12885-015-1404-9) contains supplementary material, which is available to authorized users.

## Background

Colorectal cancer is the third most common cancer and a major cause of morbidity and mortality worldwide [1, 2]. With 55,000 to 60,000 newly diagnosed cases each year, colorectal cancer ranks second among all cancer types in Germany, and with more than 1 million new cases it ranks third on a worldwide basis [3]. Advanced therapy regimens and prevention programs in the last few decades have definitely reduced mortality rates and improved outcomes [4, 5]. Adjuvant chemotherapy was found to enhance survival after curative surgery in patients with lymph node-positive colon cancer. The MOSAIC trial, a milestone publication [6], established the combination of 5-fluorouracil (5-FU), leucovorin and oxaliplatin (FOLFOX4) as the standard adjuvant chemotherapy regimen, replacing 5-FU and leucovorin in UICC-stage III colon cancer-patients after R0 resection [6, 7].

However, the benefit of adjuvant therapy after R0 resection of colon cancer in UICC stage II remains unclear. Summarizing several large studies and meta-analyses of patients with stage II colon cancer, adjuvant therapy did not prove beneficial in this group. Therefore, adjuvant treatment is not routinely recommended in these patients [8]. However, there is an ongoing discussion as to whether adjuvant chemotherapy may be beneficial in a subgroup of UICC II patients in a “high-risk situation”, such as those with large tumors (T4) [9]. Therefore, despite the paucity of appropriate data, the German clinical practice guidelines for CRC state that adjuvant chemotherapy may be considered in these patients.

One reason for recommending adjuvant chemotherapy in T4 UICC II tumors is the distinct biological behavior of these cancers and their significantly poorer prognosis. Regrettably, the potential benefit of adjuvant treatment in patients with large (T4) tumors has not been specifically analyzed in any of the studies addressing the role of adjuvant chemotherapy in patients with UICC II stage colon cancer [10–12].

Therefore, we investigated an East-Bavarian cohort of 1937 patients who had undergone curative resection of UICC II CRC in regard of the potential benefit of adjuvant chemotherapy in patients with T4 tumors. The results of our retrospective study point towards a significant benefit of adjuvant treatment in these patients, and may help to solve the current dilemma of deficient data on the treatment of T4 UICC II colon cancer.

## Methods

### Study design

We performed a retrospective analysis of clinical data obtained from the population-based cancer register at the Regensburg Tumor Center in Eastern Bavaria, Germany. Survival and recurrence rates were investigated in a cohort of patients with R0-resected colon cancer, TNM stage T4N0M0, diagnosed between 2002 and 2012. Patients receiving adjuvant chemotherapy were compared to those who had received no chemotherapy. Data collection and the retrospective analysis of patient information were anonymized in accordance with the Declaration of Helsinki, and approved by the Bavarian Law of Cancer Registration; the patients’ written informed consent was not required.

### Background and data collection

The Regensburg Tumor Center was founded in 1991. The cancer registry includes epidemiological and clinical data from all consenting patients with malignancies diagnosed and treated by specialists as well as general practitioners in the districts of Oberpfalz and Niederbayern in Eastern Bavaria. The region covered by the center consists of about 2.1 million inhabitants. More than 1000 practicing doctors, the University Hospital of Regensburg, and 53 regional hospitals were involved in the area-wide, population-based, cross-sector documentation of cancer patients. The registry receives medical information from all regional pathologists and clinicians at the time of diagnosis, treatment, and during follow-up. Physicians may enter the data in case forms, use computer-assisted tumor documentation, or send medical reports to the registry. At the office of the registry the data are extracted, recorded, and fed into a central database by suitably trained personnel. The patients’ living status and disease recurrence are ascertained from clinical reports, death certificates issued by the local public health departments, and the registration offices of the respective residential districts. Data are processed and secured according to the Bavarian Law of Cancer Registries. According to the estimates of the German Robert-Koch institute (RKI), the Regensburg Tumor Center includes more than 90 % of the estimated number of cancer cases. Thus, the data were comprehensive and selection bias was largely excluded.

The baseline cohort of the present study consisted of patients with the ICD-10-GM (https://www.dimdi.de/static/de/klassi/icd-10-gm) diagnosis C18, i.e. a malignant neoplasm of colon. Patients with histologically confirmed adenocarcinoma from 2002 to 2012 were included in the study (Additional file 1: Fig. S1). Neuroendocrine tumors were excluded. The TNM stage according to the UICC classification of malignant tumors was IIB, corresponding to T4N0M0. In other words, the tumor had invaded adjacent organs or penetrated the visceral peritoneum, with no distant metastases and/or regional lymph node metastases. Only patients who had undergone surgery for pathologically confirmed total or local residual R0 disease were included. Since a preceding evaluation had revealed that chemotherapy was administered to only one patient older than 79 years of age, the analysis was confined to patients younger than 80 years at the time of diagnosis. Patients who died within 30 days after the diagnosis were excluded, as chemotherapy could not be used effectively in these patients.

### Statistical analysis

Continuous data are described as means, median, minimum, maximum values and standard deviation, and categorical data are expressed as absolute frequencies and relative percentages. Patient characteristics were compared with T-tests for continuous data, and Chi-square tests for categorical variables. Life status for estimating overall survival rates was derived from clinical reports, death certificates, and registration offices. Recurrences were derived from clinical reports and were defined as locoregional relapse and/or recurrence as distant metastases. Overall survival rates (OS), recurrence-free survival rates (RFS), and cumulative recurrence rates were analyzed from the date of diagnosis until the first event. Patients with and without adjuvant chemotherapy were compared with the Kaplan-Meier method. The follow-up period and survival times were right censored using 31 December 2012 as the cut-off date. Survival differences were tested for statistical significance by the two-sided log rank test; the level of significance was set to 0.05. To determine the influence of adjuvant chemotherapy and further covariables on overall survival, we performed univariate and multivariate regression analysis using Cox proportional hazard models. In multivariate analysis, the hazard ratio (HR) of chemotherapy versus no chemotherapy was adjusted for the covariables of age, sex, postoperative histopathological grading, and the number of lymph nodes. Again, a two-sided p-value of 0.05 was considered to indicate statistical significance. Hazard ratios and corresponding 95 % confidence intervals (CI) were calculated and considered statistically significant when the CI excluded 1.0.

All analyses were performed using IBM SPSS Statistics, version 21.0.

## Results

### Characterization of patients with and without adjuvant chemotherapy for T4 UICC II colon cancer

Of 240 patients with T4 UICC II colon cancer, 79 had received adjuvant treatment after surgery (32.9 %); their mean age was 63.9 years (median, 66.5 years). Patients who received no adjuvant chemotherapy were on average 68.3 years old (median 69.5, *p* = 0.002). The large majority of patients had cancer of the sigmoid colon (30.4 %). The overall number of patients with cancers in the various locations of the colon were similar in those with and without adjuvant chemotherapy. The cancer was located more frequently in the sigmoid colon in patients who received adjuvant therapy; the difference was not significant (*p* = 0.258). The grading of tumors was similar in the two groups (*p* = 0.254). Besides, a larger number of patients who had received adjuvant treatment were alive at the time of data analysis (82.3 % vs. 65.2 %, *p* = 0.006, Table 1). The mean duration of follow-up was 47.2 months (median 48.2 months).

**Table 1:** Clinical characteristics of patients with T4 UICC II colon cancer. Patients over 80 years of age and those who died within 30 days after the diagnosis were excluded

Chemotherapy								
		+ CTX		− CTX		Total		Chi square
		N	%	N	%	N	%	*p*-value
Sex	male	47	59.5 %	97	60.2 %	144	60.0 %	0.910697
	female	32	40.5 %	64	39.8 %	96	40.0 %	
Age at diagnosis	30–39	2	2.5 %	1	0.6 %	3	1.3 %	
	40–49	7	8.9 %	9	5.6 %	16	6.7 %	
	50–59	16	20.3 %	14	8.7 %	30	12.5 %	0.030225
	60–69	28	35.4 %	61	37.9 %	89	37.1 %	
	70–79	26	32.9 %	76	47.2 %	102	42.5 %	
Diagnosis ICD-10	C18.0 caecum	14	17.7 %	31	19.3 %	45	18.8 %	
	C18.1 appendix	3	3.8 %	5	3.1 %	8	3.3 %	
	C18.2 ascending colon	14	17.7 %	23	14.3 %	37	15.4 %	
	C18.3 hepatic flexure	2	2.5 %	14	8.7 %	16	6.7 %	
	C18.4 transverse colon	5	6.3 %	18	11.2 %	23	9.6 %	0.258347
	C18.5 splenic flexure	3	3.8 %	11	6.8 %	14	5.8 %	
	C18.6 descending colon	5	6.3 %	11	6.8 %	16	6.7 %	
	C18.7 sigmoid colon	32	40.5 %	41	25.5 %	73	30.4 %	
	C18.8 overlapping sites of colon	1	1.3 %	6	3.7 %	7	2.9 %	
	C18.9 colon. unspecified	0	0.0 %	1	0.6 %	1	0.4 %	
Grading	2	56	70.9 %	125	77.6 %	181	75.4 %	0.253519
	3	23	29.1 %	36	22.4 %	59	24.6 %	
Lymphnodes examined	1–11	4	5.1 %	11	6.8 %	15	6.3 %	
	12–23	44	55.7 %	95	59.0 %	139	57.9 %	0.806346
	24+	30	38.0 %	52	32.3 %	82	34.2 %	
unknown	1	1.3 %	3	1.9 %	4	1.7 %		
Lymph vessel invasion	L0	30	38.0 %	62	38.5 %	92	38.3 %	0.758440
	L1	37	46.8 %	69	42.9 %	106	44.2 %	
	LX	12	15.2 %	30	18.6 %	42	17.5 %	
Vein invasion	V0	53	67.1 %	92	57.1 %	145	60.4 %	0.068554
	V1	12	15.2 %	18	11.2 %	30	12.5 %	
	VX	14	17.7 %	51	31.7 %	65	27.1 %	
Recurrence	no	63	79.7 %	138	85.7 %	201	83.8 %	0.238964
	yes	16	20.3 %	23	14.3 %	39	16.3 %	
Locoregional recurrence	no	72	91.1 %	154	95.7 %	226	94.2 %	0.160988
	yes	7	8.9 %	7	4.3 %	14	5.8 %	
Distant recurrence	no	67	84.8 %	146	90.7 %	213	88.8 %	0.176026
	yes	12	15.2 %	15	9.3 %	27	11.3 %	
Vital status	alive	65	82.3 %	105	65.2 %	170	70.8 %	0.006285
	dead	14	17.7 %	56	34.8 %	70	29.2 %	
Death or recurrence	no	58	73.4 %	95	59.0 %	153	63.8 %	0.029079
	yes	21	26.6 %	66	41.0 %	87	36.3 %	
	Total	79	100.0 %	161	100.0 %	240	100.0 %	

### Differences in overall and recurrence-free survival in T4 UICC II patients with or without adjuvant chemotherapy

**Fig. 1:**
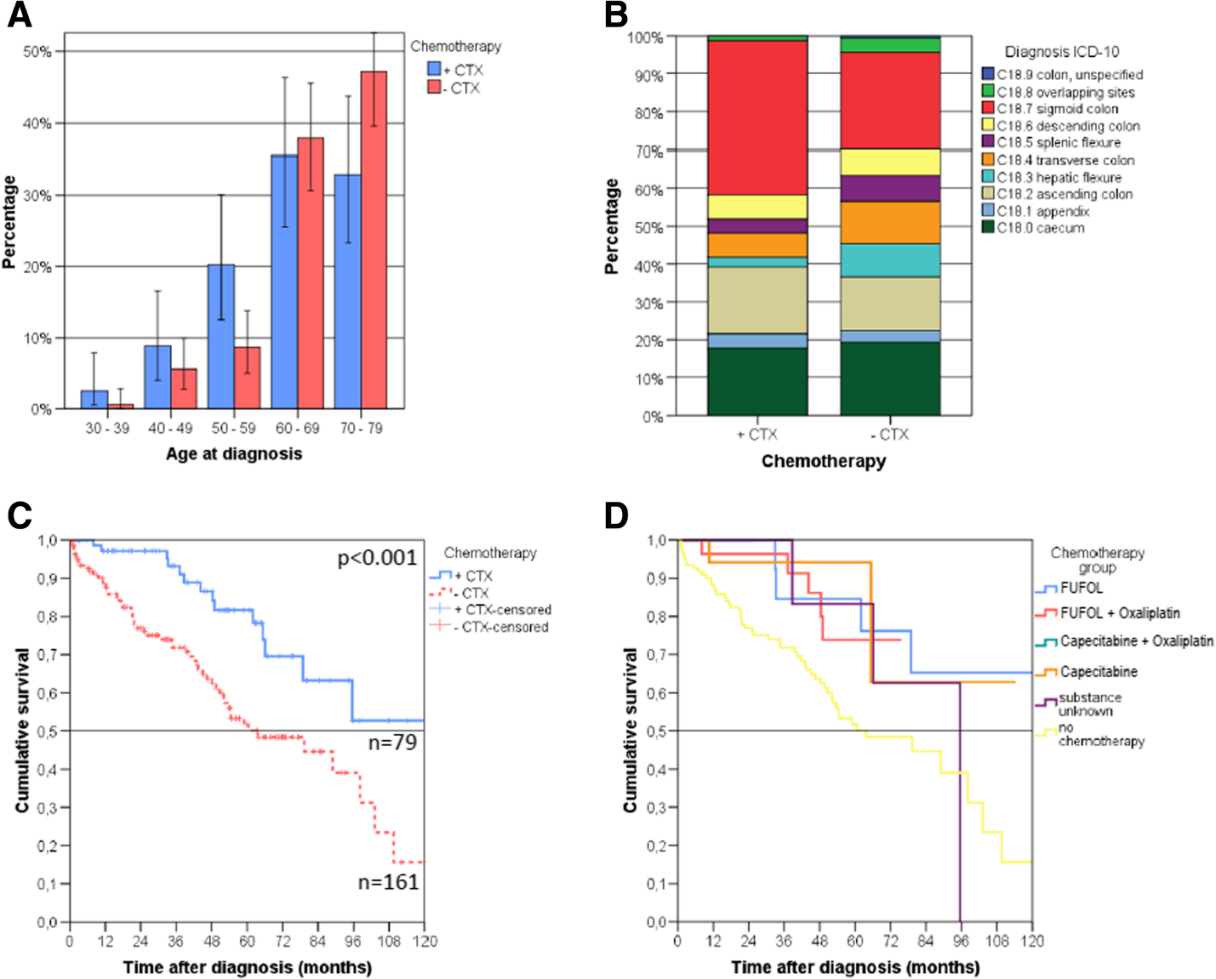
**a.** ‘A larger number of younger patients with T4 UICC stage II received adjuvant chemotherapy. **b.** The location of disease did not differ significantly between treated and untreated patients. **c.** Overall survival benefit for T4 UICC II colon cancer patients who received adjuvant chemotherapy (*p* < 0.001). **d.** All of the of adjuvant treatments used (5-Fluorouracil and folinic acid (FUFOL), FUFOL and oxaliplatin, Capecitabin and Oxaliplatin, and Capecitabine) appear to be beneficial compared to patients not receiving adjuvant chemotherapy. Patients over 80 years of age and those who died within 30 days after the diagnosis were excluded

We investigated differences in overall survival in relation to adjuvant chemotherapy in T4 UICC II colon cancer patients. Overall survival improved significantly in patients who received adjuvant therapy (*p* < 0.001), with 5-year survival rates of 81.7 % vs. 51.7 % (Fig. 1, Table 2). Cumulative overall recurrence rates in the former group were slightly higher but did not differ significantly (*p* = 0.700, Table 2, Additional file 2: Fig. S2, Additional file 3: Table S1); nor did the rates estimated separately for locoregional recurrences and distant metastases (Additional file 4: Table S2). Kaplan-Meier estimates of recurrence-free survival yielded a 5-year survival rate of 68.2 % in treated, versus 45.9 % in untreated patients, showing the significant benefit of adjuvant chemotherapy (*p* = 0.008. Table 2, Additional file 2: Fig. S2, Additional file 5: Table S3).

**Table 2:** Overall survival rates OAS (Kaplan-Meier) of patients with T4 UICC II colorectal cancer. Patients over 80 years of age were excluded

Group	Chemotherapy	Number		Overall survival rate OAS		Log-Rank
		Total	Events	5 years (%)	Median OAS (m)	*P*-value
All patients (deceased within 30 days excluded)	+ CTX	79	14	81.7	−	<0.001
	−CTX	161	56	51.7	63.3	
Deceased within 60 days excluded	+ CTX	79	14	81.7	−	0.002
	−CTX	156	51	53.7	79.3	
Deceased within 90 days excluded	+ CTX	79	14	81.7	95.5	0.006
	−CTX	152	47	55.4	79.3	
Age at diagnosis < 60 years	+ CTX	25	5	90.9	−	
	−CTX	24	4	70.0	−	0.726
Age at diagnosis 60 − 69 years	+ CTX	28	4	77.8	−	0,011
	−CTX	61	25	44.9	53.5	
Age at diagnosis 70–79 years	+ CTX	26	5	77.0	–	0.064
	−CTX	76	27	52.0	63.6	
Tumor grading G2	+ CTX	56	8	87.0	−	0.009
	−CTX	125	39	60.0	89.0	
Tumor grading G3	+ CTX	23	6	68.9	95.5	0.014
	−CTX	36	17	23.4	42.9	

The results of the Kaplan-Meier estimates were confirmed by Cox proportional hazard analyses. In univariate and multivariate models (adjusted for age at diagnosis, sex, grading, number of examined lymph nodes, lymph vessel invasion, vein invasion), adjuvant chemotherapy persisted as an independent significant factor influencing overall survival (adjusted HR 0.41, *p* = 0.001) and recurrence-free survival (adjusted HR = 0.55, *p* = 0.010). Of the studied variables, only the number of examined lymph nodes (*p* < 0.001) and tumor grading (*p* = 0.014) remained independent factors besides chemotherapy (Table 3).

**Table 3:** Multivariate Cox regression for overall survival in patients with R0 resected T4 UICC II colorectal cancer. Patients over 80 years of age were excluded

Variable	Value	p value	Hazard Ratio	lower 95 % CI	upper 95 % C
Chemotherapy	−CTX		1.00		
	+CTX	0.005	.041	0.22	0.76
Age at diagnosis (continuous)		0.086	1.03	0.99	1.06
Sex	Male		1.00		
	Female	0.275	0.75	0.44	1.26
Grading	G2		1.00		
	G3	<0.001	2.64	1.50	4.64
Lymphnodes examined	1–11		1.00		
	12–23	0.003	0.31	0.15	0.68
	24+	0.003	0.26	0.11	0.64
	Unknown	0.503	0.62	0.16	2.50
Lymph vessel invasion	LO		1.00		
	L1	0.573	1.22	0.62	2.39
	LX	0.114	0.41	0.41	1.24
Vein invasion	VO		1.00		
	V1	0.714	0.86	0.37	1.97
	VX	0.054	2.10	0.99	4.48

In order to fully estimate the impact of adjuvant chemotherapy in T4 UICC II patients, we initially excluded patients who died within 30 days after surgery. These patients were likely to have died due to non-cancer-related reasons. Besides, chemotherapy could not be effectively applied or develop its potential in this group. The significant benefit of adjuvant chemotherapy in regard of overall survival persisted when the additional sub-analyses excluded patients who died within 60 and 90 days after diagnosis, all of them occurring in the non-treatment group (Table 2, Additional file 6: Fig. S3).

### Survival benefit in relation to the drugs administered as adjuvant treatment for T4 UICC II cancer

Once the decision to administer adjuvant treatment has been made, the choice of drugs needs to be determined. Specifically, one needs to decide whether to use 5-fluorouracil alone or with additional oxaliplatin. We divided our T4 UICC II patients who had received adjuvant chemotherapy into additional subgroups, depending on the drugs they had received. The three groups, i.e. FUFOL (*n* = 15), FUFOL + oxaliplatin (*n* = 27), and “other substances” (*n* = 22), contained more than 10 patients each and were therefore considered for individual analyses; 161 patients remained untreated. As the numbers of patients in the three subgroups remained low, the subgroup analyses must be considered exploratory. However, patients receiving treatment showed a significant benefit in terms of overall survival (*p* = 0.041 for FUFOL, *p* = 0.047 for FUFOL + oxaliplatin, and *p* = 0.037 for “other drugs”, Fig. 1d, Table 4, Additional file 7: Table S4) compared to untreated patients. Thus, the choice of drugs seemed to be of secondary importance for the achievement of a survival benefit.

**Table 4:** Statistical evaluation of the benefit of diverse chemotherapy regimens used for adjuvant treatment in patients with T4 UICC II

Group comparisons													
		FUFOL		FUFOL + Oxaliplatin		Others + Oxaliplatin		Other substances		Substance unknown		No chemotherapy	
	Chemotheraphy	Chi-		Chi-		Chi-		Chi-		Chi-		Chi-	
	group	square	Sig.	square	Sig.	square	Sig.	square	Sig.	square	Sig.	square	Sig.
Long Rank	FUFOL			,072	,788	.	.	,013	,909	,563	,453	4,179	,041
(Mantel-Cox)	FUFOL + Oxaliplatin	,072	,788			,074	,785	,192	,661	,015	,901	3,934	,047
	Others + Oxaliplatin	.	.	,074	,785			,118	,732	.	.	,628	,428
	Other substances	,013	,909	,192	,661	,118	,732			,223	,636	4,370	,037
	Substance unknown	,563	,453	,015	,901			,223	,636			,681	,409
	No chemotheraphy	4,179	,041	3,934	,047	,628	,428	4,370	,037	,681	,409		

### Influence of tumor grading on survival in T4 UICC II cancers

**Fig. 2:**
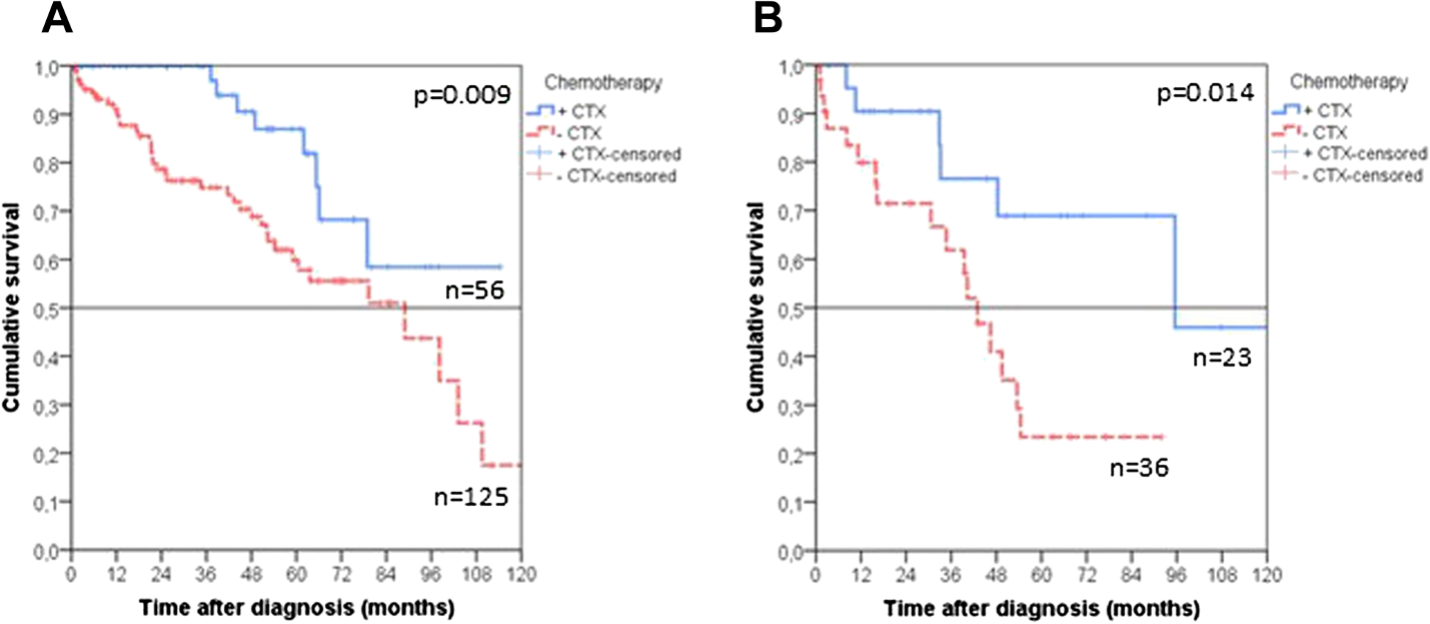
Kaplan Meier estimates for overall survival in patients with R0 resected T4 UICC II colorectal cancer, depending on tumor grading. Patients with G2 (**a**, *p* = 0.009) and G3 (**b**, *p* = 0.014) tumors benefited significantly from adjuvant chemotherapy

In order to determine a potential indication for adjuvant chemotherapy, patients were additionally stratified on the basis of tumor grading. T4 UICC II patients with G3 cancers benefited to a significantly greater extent from adjuvant chemotherapy (5-year OS 68.9 % vs. 23.4 %, *p* = 0.014, Table 2, Fig. 2). Furthermore, survival was significantly improved in G2 T4 UICC II tumors (5-year OS 87.0 % vs. 60.0 %, *p* = 0.009, Table 2, Fig. 2).

### No influence of age at diagnosis on survival in T4 UICC II CRC patients

Besides tumor grading, the influence of the patient’s age at diagnosis on survival was investigated in T4 UICC II patients with colon cancer. In the small number of patients younger than 60 years of age, we observed a slight but non-significant trend in favor of adjuvant chemotherapy (*p* = 0.726). In contrast, an additional survival benefit due to adjuvant chemotherapy was observed in patients aged 60 to 69 years and those between 70 and 79 years, the difference being significant only in the younger age group [*p* = 0.011 (60–69 years); *p* = 0.064 (70–79 years), Additional file 8: Fig. S4].

## Discussion

Generally, the prognosis of disease is considered favorable in patients with UICC stage II colon cancer who have undergone R0 resection; adjuvant therapy is currently not recommended in these patients. The absolute benefit of adjuvant therapy in patients with UICC stage II CRC without additional risk factors ranges between 2 % and 5 %. Overall however, studies and meta-analyses in patients with stage II colon cancer revealed no significant survival advantage [10, 11, 13, 14]. A pooled analysis of seven randomized trials comparing adjuvant chemotherapy with surgery alone showed, in univariate analysis, a significant improvement of 5-year disease-free survival (DFS) (72 % versus 76 %; *p* = 0.049), but not 5-year overall survival (80 % versus 81 %; *p* = 0.1127). However, the therapy regimens varied significantly in these studies and the majority of them consisted of small numbers of patients. In contrast, adjuvant treatment was found to yield a significant benefit in the QUASAR study [12], which is the largest study investigating the role of adjuvant chemotherapy in UICC II cancer. In view of these controversial data, the potential benefit of adjuvant chemotherapy for specific subgroups of patients with UICC II CRC and especially those with additional risk factors is still controversially discussed. [12] These risk factors include lymph node sampling below 12, a poorly differentiated tumor, vascular or lymphatic or perineural invasion, a pT4 stage, and intestinal occlusion or perforation on clinical investigation [9]. Although several authors and reviews have suggested adjuvant therapy for these patients, we lack data concerning the benefit of adjuvant therapy in this subgroup. Furthermore, once the decision in favor of adjuvant treatment has been made, the choice of drugs - 5-fluorouracil with or without oxaliplatin – is a debated issue. Data from the MOSAIC [6] and the NSABP protocol C05-C08 trials indicated a borderline benefit of 2–3 % in 5-year OS rates when using additional oxaliplatin [15]. Therefore, we assessed the benefit of adjuvant chemotherapy in a large cohort of T4 UICC II patients within a specific region of Germany.

Among our patients with T4 UICC II tumors, those who received chemotherapy were significantly younger (*p* = 0.002) and had a (non-significant) larger number of G3 tumors (*p* = 0.286). This difference between treated and untreated patients may assist the clinician in selecting patients for adjuvant chemotherapy. Since patients over 80 years of age rarely received adjuvant chemotherapy, we excluded these patients from analysis. For all others, the difference in age at diagnosis may potentially influence patient survival because older patients may have more severe co-morbidities. Therefore we performed separate survival analyses in the age groups <60 years, 60–69 years, and 70–79 years at diagnosis. The difference in survival could also be observed in the 60- to 69-year and 70- to 79-year-old groups. Among patients under 60 years of age, we noted a slight benefit of adjuvant chemotherapy. However, this group consisted of a small number of patients. Besides, the significant results of the univariate Cox regression analysis persisted in a multivariate regression analysis with adjustment for age at diagnosis.

Patients with perioperative mortality are less likely to receive adjuvant chemotherapy, but may have shorter survival times. To rule out the influence of perioperative mortality or even mortality on our analysis, we excluded patients with events within the first 30, 60, and 90 days after diagnosis. All three analyses showed a significant benefit for patients treated with adjuvant chemotherapy (Fig. 1, Additional file 8: Fig. S4). Thus, perioperative mortality had no impact on the analysis. Although the cumulative rates for recurrent events were slightly but non-significantly higher among patients who received adjuvant chemotherapy, the benefit persisted in this group in respect of recurrence-free survival.

Drawback of our approach was that the reports did not contain details about side effects under treatment. However, all chemotherapy regimens used for adjuvant treatment were well established drug combinations routinely used in UICC stage III CRC. Therefore, we considered all of the employed chemotherapy regimens as feasible and generally well tolerated.

Overall, our data demonstrated the significant benefit of adjuvant chemotherapy in patients with T4 UICC II colon cancer. Since our findings suggested a change in the current treatment recommendations, we performed further subgroup analyses excluding the influence of age or perioperative morbidity. Based on our data we conclude that adjuvant chemotherapy is justified in T4 UICC II patients. Further prospective studies should be performed to obtain confirmation of this thesis.

## Conclusion

A retrospective investigation showed the significant benefit of adjuvant chemotherapy in T4 UICC II patients. Based on our data, adjuvant chemotherapy should be seriously considered in T4 UICC II patients with colon cancer. Tumor grading may also influence the benefit of adjuvant treatment and warrants further investigation.

## Additional files

Additional file 1: Figure. S1. Patients with T4 UICC II and adjuvant treatment, separated per year between 2002 and 2012.
Additional file 2: Figure. S2. Comparison of cumulative 10-year recurrence rates in patients with and without adjuvant chemotherapy after R0 resection of T4 colon cancer (Kaplan-Meier, all patients, *p* = 0.700).
Additional file 3: Table S1. Recurrence-free survival rates RFS (Kaplan-Meier) of patients with T4 UICC II colorectal cancer. Patients over 80 years were excluded.
Additional file 4: Table S2. Cumulative recurrence rates (Kaplan-Meier) of patients with T4 UICC II colorectal cancer. Patients over 80 years were excluded.
Additional file 5: Table S3. Cox-Regression for several outcomes of chemotherapy yes versus no, unadjusted and adjusted (adjusted for age at diagnosis, sex, grading, examined lymph nodes, lymphatic vessel invasion, vein invasion). Patients over 80 years were excluded.
Additional file 6: Figure. S3. Recurrence-free survival of patients in relation to adjuvant chemotherapy (A), and overall survival in a subgroup, excluding all events 90 days after surgery (B): the significant benefit of adjuvant chemotherapy was noted in both groups.
Additional file 7: Table S4. Listing of additional chemotherapies after recurrence of disease.
Additional file 8: Figure. S4. Survival of patients with T4 UICC II tumors and different ages at diagnosis. Among patients younger than 60 years (A), we observed a trend in favor of chemotherapy. However, the group consisted of a small number of patients. A significant difference in favor of chemotherapy was noted among patients aged 60–69 years at diagnosis (B), and a marginally non-significant difference in patients aged 70–79 years (C).
