# Supplementary figures and images for: Benefit of adjuvant chemotherapy in patients with T4 UICC II colon cancer
Source: BMC Cancer. 2015 May 20;15:419. doi: 10.1186/s12885-015-1404-9 (PMC4451874; doi:10.1186/s12885-015-1404-9)

**Suppl. Figure 1**

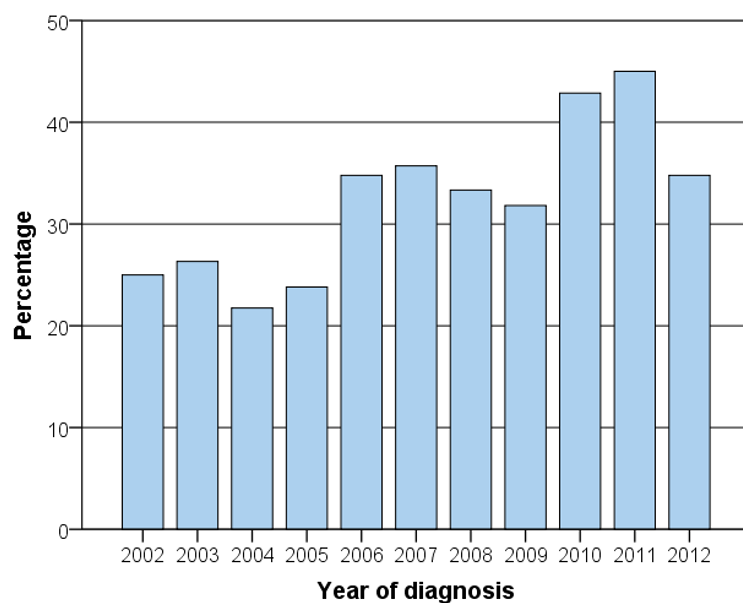

|                   |       | Chemotherapy |       |        |       |        |        |
|-------------------|-------|--------------|-------|--------|-------|--------|--------|
|                   |       | yes          |       | no     |       | total  |        |
|                   |       | number       | %     | number | %     | number | %      |
| Year of diagnosis | 2002  | 3            | 25,0% | 9      | 75,0% | 12     | 100,0% |
|                   | 2003  | 5            | 26,3% | 14     | 73,7% | 19     | 100,0% |
|                   | 2004  | 5            | 21,7% | 18     | 78,3% | 23     | 100,0% |
|                   | 2005  | 5            | 23,8% | 16     | 76,2% | 21     | 100,0% |
|                   | 2006  | 8            | 34,8% | 15     | 65,2% | 23     | 100,0% |
|                   | 2007  | 10           | 35,7% | 18     | 64,3% | 28     | 100,0% |
|                   | 2008  | 7            | 33,3% | 14     | 66,7% | 21     | 100,0% |
|                   | 2009  | 7            | 31,8% | 15     | 68,2% | 22     | 100,0% |
|                   | 2010  | 12           | 42,9% | 16     | 57,1% | 28     | 100,0% |
|                   | 2011  | 9            | 45,0% | 11     | 55,0% | 20     | 100,0% |
|                   | 2012  | 8            | 34,8% | 15     | 65,2% | 23     | 100,0% |
|                   | total | 79           | 32,9% | 161    | 67,1% | 240    | 100,0% |

Supplement: Additional file 1: Figure. S1. — Patients with T4 UICC II and adjuvant treatment, separated per year between 2002 and 2012. [file 12885_2015_1404_MOESM1_ESM.pdf]

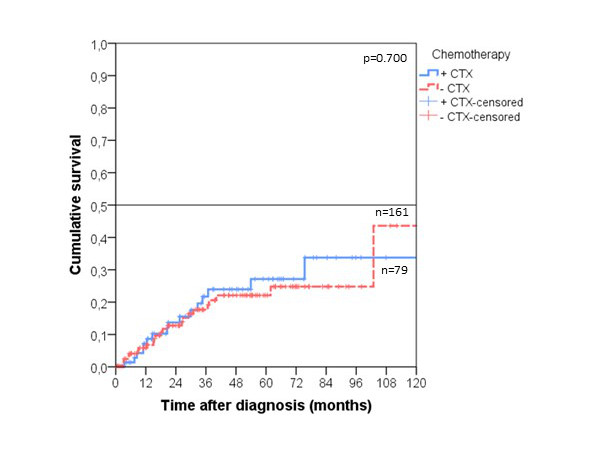

Supplement: Additional file 2: Figure. S2. — Comparison of cumulative 10-year recurrence rates in patients with and without adjuvant chemotherapy after R0 resection of T4 colon cancer (Kaplan-Meier, all patients, p = 0.700). [file 12885_2015_1404_MOESM2_ESM.jpeg]

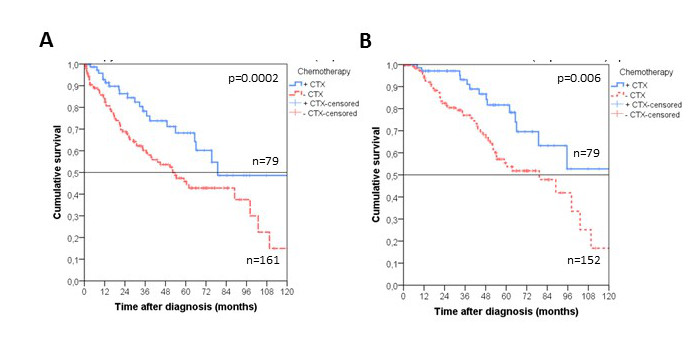

Supplement: Additional file 6: Figure. S3. — Recurrence-free survival of patients in relation to adjuvant chemotherapy (A), and overall survival in a subgroup, excluding all events 90 days after surgery (B): the significant benefit of adjuvant chemotherapy was noted in both groups. [file 12885_2015_1404_MOESM6_ESM.jpeg]

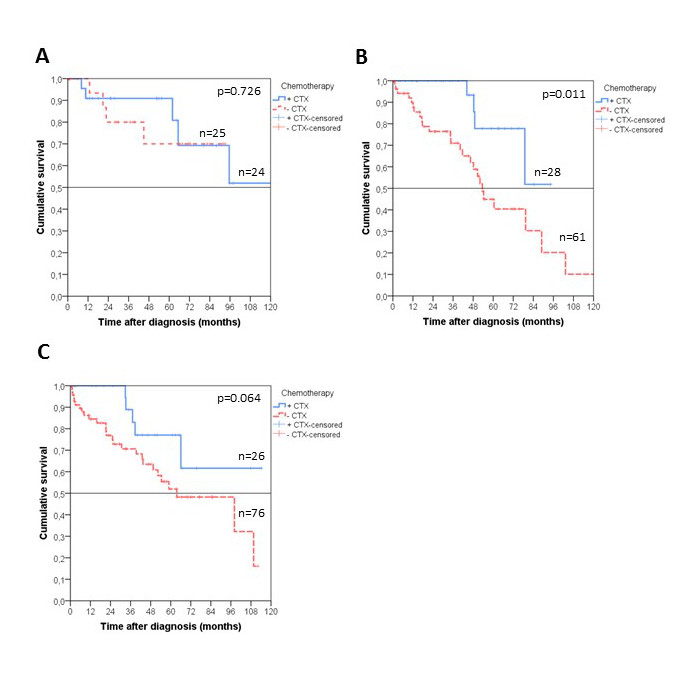

Supplement: Additional file 8: Figure. S4. — Survival of patients with T4 UICC II tumors and different ages at diagnosis. Among patients younger than 60 years (A), we observed a trend in favor of chemotherapy. However, the group consisted of a small number of patients. A significant difference in favor of chemotherapy was noted among patients aged 60–69 years at diagnosis (B), and a marginally non-significant difference in patients aged 70–79 years (C). [file 12885_2015_1404_MOESM8_ESM.jpeg]
