# Supplementary material for: Benefit of adjuvant chemotherapy in patients with T4 UICC II colon cancer
Source: BMC Cancer. 2015 May 20;15:419. doi: 10.1186/s12885-015-1404-9 (PMC4451874; doi:10.1186/s12885-015-1404-9)
Supplement: Additional file 3: Table S1. — Recurrence-free survival rates RFS (Kaplan-Meier) of patients with T4 UICC II colorectal cancer. Patients over 80 years were excluded. [file 12885_2015_1404_MOESM3_ESM.pdf]

Group	Chemotherapy	Number		Recurrence free survival rate RFS		Log-Rank
		Total	Events	5 years (%)	Median RFS (m)	P-value
All patients	+ CTX<br>- CTX	79<br>161	21<br>66	68.2<br>45.9	78.9<br>52.4	0.008
