# Supplementary material for: Benefit of adjuvant chemotherapy in patients with T4 UICC II colon cancer
Source: BMC Cancer. 2015 May 20;15:419. doi: 10.1186/s12885-015-1404-9 (PMC4451874; doi:10.1186/s12885-015-1404-9)
Supplement: Additional file 4: Table S2. — Cumulative recurrence rates (Kaplan-Meier) of patients with T4 UICC II colorectal cancer. Patients over 80 years were excluded. [file 12885_2015_1404_MOESM4_ESM.pdf]

Group	Chemotherapy	Number		Cumulative recurrence rate		Log-Rank
		Total	Events	5 years (%)	Median (m)	P-value
All recurrences	+ CTX	79	16	27.1	-	0.700
	- CTX	161	23	22.1	-	
Locoregional recurrences	+ CTX	79	7	10.1	-	0.367
	- CTX	161	7	6.3	-	
Distant recurrences	+ CTX	79	12	21.7	-	0.508
	- CTX	161	15	13.6	-	
