# Supplementary material for: Benefit of adjuvant chemotherapy in patients with T4 UICC II colon cancer
Source: BMC Cancer. 2015 May 20;15:419. doi: 10.1186/s12885-015-1404-9 (PMC4451874; doi:10.1186/s12885-015-1404-9)
Supplement: Additional file 5: Table S3. — Cox-Regression for several outcomes of chemotherapy yes versus no, unadjusted and adjusted (adjusted for age at diagnosis, sex, grading, examined lymph nodes, lymphatic vessel invasion, vein invasion). Patients over 80 years were excluded. [file 12885_2015_1404_MOESM5_ESM.pdf]

	Hazard ratio Chemotherapy yes vs no							
	**unadjusted (univariable)**				**adjusted (multivariable)**			
Outcome	p-value	**HR**	lower 95% CI	upper 95% C	p-value	**HR**	lower 95% CI	upper 95% C
Overall survival OAS	0.001	**0.38**	0.21	0.68	0.005	**0.41**	0.22	0.76
Recurrence free survival RFS	0.010	**0.52**	0.32	0.85	0.020	**0.55**	0.33	0.91
Recurrence free time	0.700	**1.13**	0.60	2.15	0.868	**0.95**	0.49	1.83
