# Supplementary material for: Benefit of adjuvant chemotherapy in patients with T4 UICC II colon cancer
Source: BMC Cancer. 2015 May 20;15:419. doi: 10.1186/s12885-015-1404-9 (PMC4451874; doi:10.1186/s12885-015-1404-9)
Supplement: Additional file 7: Table S4. — Listing of additional chemotherapies after recurrence of disease. [file 12885_2015_1404_MOESM7_ESM.pdf]

Chemotherapy	N	%
5-Fluorouracil (+/- folinic acid)[1]	8	20,5
5-Fluorouracil, folinic acid, Irinotecan (FOLIFIRI)	7	17,9
5-Fluorouracil, folinic acid, Oxaliplatin (FOLIFOX)[2]	5	12,8
5-Fluorouracil, folinic acid, Irinotecan (FOLIFIRI) + Bevacizumab	2	5,1
5-Fluorouracil, folinic acid, Oxaliplatin (FOLIFOX) + Bevacizumab	2	5,1
Cetuximab	1	2,6
Chemotherapy not specified	1	2,6
No Chemotherapy	13	33,3
Total	39	100

[1] including treatment with Capecitabin, [2] including FOLFOX 4, 6, 7 and XELOX regimens
